# HIV/AIDS and viral hepatitis B/C co-infection during pregnancy

**DOI:** 10.1186/1471-2334-13-S1-P23

**Published:** 2013-12-16

**Authors:** Carmen Georgescu, Mihai Mitran, Doru Pană, Loredana Mitran

**Affiliations:** 1 Clinical Hospital of Obstetrics and Gynecology “Prof. Dr. Panait Sârbu”, Bucharest, Romania; 2 Elias University Emergency Hospital, Bucharest, Romania

## 

This co-infection is particularly serious in pregnancy through the following consequences: coagulation disorders caused by both diseases, as well as by the antiretroviral treatment (by pancytopenia, thrombocytopenia), and modification of uterine contractility through the intervention in contractile protein metabolism and secondary anemia, found in all cases.

Association with hepatic cytolysis and hepatocellular failure is the rule. In this association, hysterectomy necessary for hemostasis in some cases was imperative.

In our practice, we have encountered the HIV-hepatitis association in 30% of cases.

At birth, HIV/AIDS and hepatitis co-infection is a major risk factor for the mother, requiring total hysterectomy for hemostasis in 16.6% of the cases.

